# Integrated transcriptomics and metabolomics analysis reveal the regulatory mechanisms underlying the combined effects of heat and glucose starvation on carotenoid biosynthesis in *Rhodotorula glutinis* YM25079

**DOI:** 10.1186/s13068-025-02678-7

**Published:** 2025-07-10

**Authors:** Xingyu Huang, Caina Guo, Xiaolan Huang, Meixia He, Jingdie Fan, Yuan Chen, Jingwen Qiu, Qi Zhang

**Affiliations:** Faculty of Life Science and Technology, Kunming University of Science and Technology, Kunming, 650500 Yunnan People’s Republic of China

**Keywords:** *Rhodotorula glutinis*, Carotenoids, Combined stress between heat and glucose starvation, Transcriptome and metabolome

## Abstract

*Rhodotorula glutinis* is an important oleaginous yeast that can synthesize various valuable compounds, including carotenoids, lipids, and exopolysaccharides. The effect of combined heat stress and glucose starvation on carotenoid biosynthesis in *R. glutinis* was investigated in this study. Carotenoid production in *R. glutinis* was promoted by heat stress, and this effect was further enhanced when glucose starvation was applied to the strain. The results of multiomics analysis revealed that the effects of heat stress and glucose starvation on promoting carotenoid biosynthesis appeared to be additive, with the combined stress leading to a further increase in reactive oxygen species (ROS) levels and a reduction in enzymatic antioxidant capacity, while carotenoid biosynthesis was prioritized simultaneously. The key responses of *R. glutinis* to combined stress include the regulation of the cell cycle and energy metabolism, maintenance of membrane integrity, an increase in ROS scavenging capacity, and non-enzymatic antioxidant activity. Additionally, several candidate genes and metabolites associated with the combined stress response were identified. To summarize, we provided new insights into optimizing fermentation processes for increased carotenoid production in *Rhodotorula glutinis* and established a molecular basis for further genetic engineering to increase carotenoid yield.

**Supplementary Information:**

The online version contains supplementary material available at 10.1186/s13068-025-02678-7.

## Introduction

Carotenoids are a class of isoprenoid compounds that are found in all photosynthetic organisms and some non-photosynthetic species [1]. They are widely used as food additives and have significant health benefits, including serving as a source of vitamin A, boosting immunity, and reducing the risk of cardiovascular disease and certain types of cancer [2, 3]. Owing to these characteristics, carotenoids have attracted considerable attention in various industries and have significant commercial value [4]. Compared to natural extraction, microbial fermentation offers a greater diversity of carotenoid structures and better control, efficiency, and sustainability, making it a more common and viable approach for industrial production [5]. However, the industrial fermentation process is complex and involves dynamic changes in various environmental factors. Oleaginous red yeasts are particularly affected by these environmental conditions, including carbon source depletion, temperature fluctuations, and variations in osmotic pressure [6]. These factors can significantly influence the growth of yeasts metabolism. Consequently, understanding and optimizing these variables has become an important area of research [7].

During the industrial production of carotenoids, strategies based on environmental stress are effective methods for enhancing carotenoid biosynthesis in microorganisms [8]. With the emergence of global warming, high temperature has become an extremely common environmental stressor and a major factor affecting the growth and development of yeast cells in nature [9]. Changing the cultivation temperature is an effective method to enhance carotenoid production, but the effect depends on the yeast strain, environmental parameters, and medium composition [10]. Increasing the cultivation temperature can significantly increase carotenoid biosynthesis in *Rhodotorula* species such as *Rhodotorula gracilis*, *Rhodotorula glutinis*, and *Rhodotorula mucilaginosa* [11, 12]. Additionally, nutrient starvation is a major environmental stressor for microorganisms, and nutrient starvation, such as nitrogen starvation, carbon starvation, and phosphorus starvation, affects carotenoid biosynthesis [13, 14]. However, these studies focused only on a single stressor. Microorganisms are often exposed to multiple stressors simultaneously in natural environments [15]. Most studies on combined stress have focused on crops [16], with limited studies addressing how yeast responds to such conditions. Therefore, elucidating the mechanisms underlying the response of yeast to combined stress is crucial for optimizing industrial fermentation processes.

Environmental stressors are usually active during microbial fermentation and significantly influence microbial physiology [17]. However, interactions among environmental stressors can amplify the adverse effects of individual stressors and may even trigger novel, unpredictable responses [18]. Studies on combined stress have revealed that crops act differently and activate distinct integrated signal networks, which can differ from responses to individual stress, especially when stress factors have antagonistic interactions [19, 20]. Compared to stressors such as high temperature, drought, and intense light that affect crops, yeasts in natural environment are more susceptible to high-temperature or nutrient starvation stress. Glucose serves as the primary carbon and energy source for yeast, and glucose starvation induced by various factors affects the growth and metabolism of yeast. Some yeast strains accumulate relatively high levels of carotenoids during periods of low carbon availability [14, 21]. Our study on *Rhodosporidium kratochvilovae* suggested that alterations in carbon metabolism pathways and an increase in the levels of reactive oxygen species (ROS) resulting from the depletion of the carbon source may explain this phenomenon [14]. While previous studies have investigated the effects of heat stress or glucose starvation on the production of carotenoids in yeast, few studies have investigated the combined effects of these two stressors and the regulatory mechanisms that promote carotenoid biosynthesis under such conditions.

*Rhodotorula glutinis* YM25079 is a cold-adapted strain isolated from the Lugu Lake area in Lijiang, Yunnan, China; it shows optimal growth at 15 °C. This strain uses various carbon sources to synthesize fatty acids, carotenoids, and extracellular polysaccharides. In another study, we investigated the regulatory mechanisms underlying carotenoid production in YM25079 after treatment with epigenetic modifiers [22]. However, the regulatory mechanisms linking environmental stress with carotenoid biosynthesis in YM25079 remain undetermined. In this study, we investigated the effects of combined heat stress and glucose starvation on carotenoid production in YM25079 through integrated transcriptomics and metabolomics and elucidated the underlying mechanisms. This study improved our understanding of the behavior of the strain under these environmental conditions and provided insights into the molecular basis to optimize fermentation processes and increase the yield of carotenoids.

## Materials and methods

### Strains and cultivation conditions

The *Rhodotorula strain* YM25079 was isolated from Lugu Lake in Lijiang, Yunnan, and identified using methods described in another study [23]. The strain was stored in YPD liquid medium (yeast extract: 10 g/L, peptone: 20 g/L, glucose: 20 g/L) supplemented with 35% glycerol and stored at –80 °C for future use. Before experimentation, the strain was inoculated in a 250 mL Erlenmeyer flask containing 50 mL of YPD liquid medium and incubated in a YRZ 201 shaker (Shanghai Xilai Biotechnology Co., Ltd.) at 160 rpm and 15 °C for 24 h. The precultured YM25079 strain (0.5 mL inoculum) was then transferred to fresh YPD liquid medium and incubated at 160 rpm under two conditions: 15 °C (control group) and 30 °C (experimental group) for 168 h. All experiments were performed in triplicate.

### Analysis of strain growth and intracellular ROS levels

The growth curve of YM25079 was plotted using the Prism 8 software by measuring the OD600 at intervals of 24 h. After 96 h, the ROS levels in the culture medium were measured using a ROS detection kit (S0033S, Shanghai Beyotime Biotechnology Co., Ltd., Shanghai, China). Fermentation broth samples were collected every 24 h, centrifuged, washed, and dried to a constant weight to determine the dry biomass. The biomass concentration (g/L) was calculated by dividing the dry biomass weight (g) by the volume of fermentation broth (L). The resulting dry biomass was ground into a powder and stored at –80 °C for subsequent experiments.

### Measurement of total lipids and carotenoids

The lipid content was determined following a previously reported method [24]. A 4 mol/L aqueous solution of hydrochloric acid was added to the dry yeast powder, followed by heating in a boiling water bath for 3 min. After cooling to room temperature, a chloroform–methanol mixture (2:1, v/v) was added, and the lower chloroform layer was collected at 4500 rpm for 5min. A 0.1% (w/v) NaCl solution was then added to the chloroform layer, and the contents were centrifuged again. Finally, the chloroform layer was evaporated to a constant weight, and the total lipid content was estimated gravimetrically.

Carotenoids were extracted following the method described in previous reports [25, 26], with slight modifications. Briefly, the yeast powder samples were ground in a quartz mortar and dissolved in acetone. The upper organic phase was collected, and the lower phase was extracted twice. The total carotenoid concentration was determined by measuring the absorbance of the acetone extract at 450 nm (OD450) using a UV–visible spectrophotometer (UV-1800PC, MAPADA, Shanghai, China), expressed as mg/g dry cell weight (DCW). The carotenoid content was calculated using the following formula: "X = 1000 EV/AW", where X represents the total carotenoid content (mg/g DCW), E represents the OD450, V represents the total volume of the acetone extract (mL), W represents the weight of the dry powder sample (g), and A represents the average extinction coefficient for carotenoids (2500).

### RNA sequencing (RNA-seq) and data analysis

Total RNA from YM25079 cells was extracted using a TRIzol® Reagent Kit (Thermo Fisher Scientific, Waltham, MA, USA) following the manufacturer’s instructions. After assessing purity, concentration, and integrity, a transcriptome library was constructed using the Illumina TruSeq™ RNA Sample Prep Kit (Illumina, San Diego, CA, USA), and sequencing was performed using the Illumina NovaSeq 6000 platform (Illumina, San Diego, CA, USA).

After evaluating the quality of the raw data, the fastp software (version 0.23.4) was used to remove adapter sequences and low-quality reads. The high-quality clean reads were aligned to the reference genome using HISAT2 (version 2.1.0) with default parameters. The expression levels of genes and transcripts were quantified using the RSEM software (version 1.42.0), and differential gene expression between samples and groups was analyzed using DESeq2. GO enrichment and statistical analysis of differentially expressed genes (DEGs) were performed using the GOATOOLS library and KOBAS software (version 3.0.3).

### Real-time quantitative PCR analysis

To evaluate the quality of the transcriptome sequencing data, RT-qPCR analysis was performed on the DEGs from the two groups. The primers used for RT-qPCR were designed using the SYBR® Green fluorescence method and NCBI Primer-BLAST (https://www.ncbi.nlm.nih.gov/tools/primer-blast/, accessed on 15 November 2023) and synthesized by Shanghai Sangon Biotech Co., Ltd. (Shanghai, China). The RNA was stored at –80 °C, and reverse transcription was performed using the HiScript II 1st Strand cDNA Synthesis Kit (+ gDNA wiper) (R212-02, Vazyme, Nanjing, China). The cDNA synthesized was used as a template for real-time quantitative PCR to compare differences in gene expression. The RT-qPCR primers used for validation are listed in Table S1.

### Metabolomics and data analysis

After fermentation, the YM25079 strain was harvested at 4500 rpm of 5min and stored at –80 °C. The samples were then transported under cold chain conditions to Shanghai Baoshu Biomedical Technology Co., Ltd. (Shanghai, China) for metabolite extraction and liquid chromatography-tandem mass spectrometry (LC–MS–MS) analysis.

Sample extraction was carried out by homogenizing accurately weighed samples in 1000 μL ice-cold 50% methanol–water and 100 mg glass beads using vortex mixing (30 s), followed by three freeze-grinding cycles. The extract was reconstituted in 300 μL ice-cold 50% methanol–water, filtered (0.22 μm), and analyzed by LC–MS [27]. LC–MS analysis was performed using a Vanquish UHPLC system (Thermo Fisher Scientific) with a Q Exactive mass spectrometer (ESI source). Separation was achieved on an ACQUITY UPLC® HSS T3 column (150 × 2.1 mm, 1.8 μm; Waters) at 40 °C with a 0.25 mL/min flow rate and 2 μL injection volume. For ESI( +), the mobile phases were 0.1% formic acid in acetonitrile (C) and in water (D); for ESI(–), acetonitrile (A) and 5 mM ammonium formate (B) were used. Both modes followed the same gradient: 0–1 min: 2% organic, 1–9 min: 2–50%, 9–12 min: 50–98%, 12–13.5 min: 98% and 13.5–14 min: 98–2%.

The raw data obtained after sequencing were converted to mzXML files using the MSConvert tool from the ProteoWizard software package (version 3.0.21229). The detection, filtering, and alignment of peaks were performed using the RxCMS package (version 3.12.0). Metabolites were identified using public databases such as the Kyoto Encyclopedia of Genes and Genomes (KEGG), LipidMaps, MassBank, and mzCloud. Pathway enrichment and topology analyses of the selected differentially expressed metabolites were conducted using the MetaboAnalyst software (version 5.0). Finally, the KEGG Mapper visualization tool was used to examine the differential metabolites and the pathway maps enriched by the identified metabolites.

### Trend analysis

Hierarchical clustering analysis of all the differentially expressed protein-coding genes and metabolites was performed using OmicShare tools (www.omicshare.com/tools). The EDG or DAM with different expression trends between the two groups will be classified into different profiles, and the P value ≤ 0.05 is set as the expression profile with significant difference.

### Protein–protein interaction (PPI) and correlation analyses of the hub genes and metabolites

The Entrez IDs of genes from each significantly enriched trend were submitted to the Search Tool for the Retrieval of Interacting Genes/Proteins (STRING) database (version 11.5) to identify genes with PPI networks. The constructed networks were then exported to Cytoscape (version 3.9.1), and the CytoHubba plugin was used to rank and classify the genes as hub genes based on degree centrality, identifying the top 20 genes with the highest interactions within their respective modules. The Pearson correlation coefficient (PCC) between genes and metabolites was calculated using the “cor” function in the R package (version R 4.3.0), and the results were visualized using the Gephi software (version 0.9.7).

## Results

### Effects of heat stress on the growth, carotenoid accumulation, and lipid production of strain YM25079

**Fig. 1:**
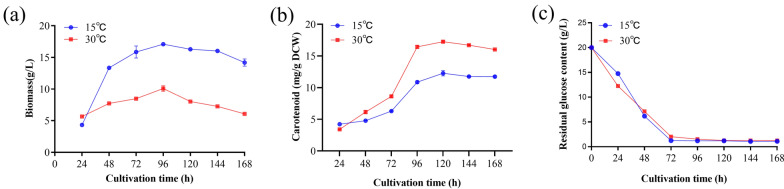
Effects of heat stress on the total biomass, carotenoid content, and residual sugars of YM25079; **a** total biomass, **b** carotenoid content, and **c** residual sugars. The data are presented as the mean ± standard deviation of triplicate samples

In this study, we evaluated the differences in the total biomass, carotenoid content, and residual sugar content of *R. glutinis* YM25079 following heat stress. The results revealed that during the early growth phase (first 24 h), the experimental group (cultivated at 30 °C) presented a slightly faster growth rate than the control group (cultivated at 15 °C), which accumulation slowed after 48 h and peaked at 96 h, with the overall biomass being lower than that of the control group (Fig. 1a). In contrast, the carotenoid content followed the opposite trend. The carotenoid content in the experimental group was higher than that in the control group at 48 h, and carotenoids began to accumulate rapidly after 72 h (Fig. 1b). Notably, carotenoid production in the experimental cohort was reduced compared to the untreated controls, primarily due to decreased biomass; however, this difference between the groups was minimal at 96 h (Figure S1a). Residual sugar measurements revealed that both groups consumed most of their glucose by 72 h and then entered a state of glucose starvation, which corresponded to the period of rapid accumulation of carotenoids (Fig. 1c). Additionally, the experimental group presented higher ROS levels (Figure S1b) and lower lipid contents during this period (Figure S1c).

### Transcriptome analysis of the YM25079 strain in response to heat stress and glucose starvation stress

To elucidate the mechanisms underlying the changes in *R. glutinis* YM25079 under heat stress and glucose starvation stress, three independent samples from the control group were selected and cultured for 36 h and 96 h (Y79_36 and Y79_96) to determine the effects of starvation stress. Three independent samples from the experimental group cultured for 96 h (Y79_96HT) were selected to assess combined stress. About 404 million raw sequencing reads were generated from the sequences of the 9 samples (Table S2). The number of raw reads per sample ranged from 42.71 million to 48.54 million, with an average of 44.98 million. After adapter sequences and low-quality reads were removed, the remaining clean reads for each sample ranged from 42.40 million to 48.34 million, with an average of 44.68 million. About 402 million clean sequencing reads were generated. These results indicated that the quality of the transcriptome sequencing data was high, with no contamination and high accuracy.

### Identification of DEGs in response to heat stress and glucose starvation stress

**Fig. 2:**
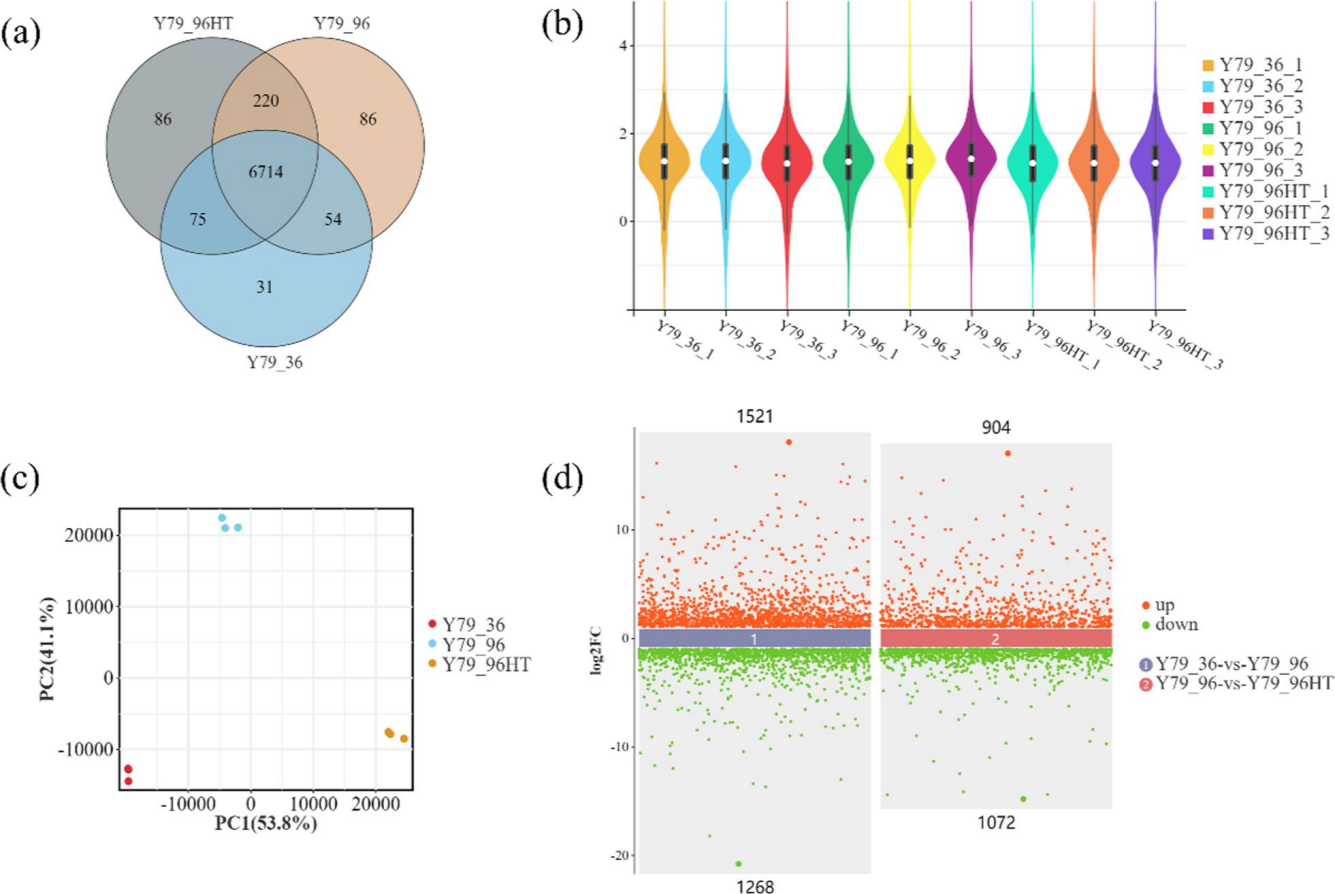
Comparative transcriptome analysis of YM25079 after heat stress and glucose starvation stress. **a** Venn diagram; **b** Violin plot; **c** PCA; **d** DEG scatter plot. Note: Y79 represents YM25079, 36 and 96 represent the culture time, and HT represents heat stress

A total of 7266 expressed genes were annotated using various databases, such as GO and KEGG, with 6714 genes shared among Y79_36, Y79_96, and Y79_96HT (Fig. 2a). The global gene expression levels were similar among the three biological replicates of each treatment (Fig. 2b), indicating good repeatability within each group. Principal component analysis (PCA) revealed that Y79_36, Y79_96, and Y79_96HT were grouped into distinct regions, highlighting the significant effect of glucose starvation and heat stress on gene expression levels in this strain (Fig. 2c). These results indicated that these genes are sufficient for subsequent differential gene expression analysis. DEGs were identified using the DESeq2 software, with an absolute log2 (fold change with FPKM) ≥ 1 used to define DEGs. Under glucose starvation, 2789 DEGs were identified (Y79_36 vs. Y79_96), of which 1268 genes were downregulated (45.46%) and 1,521 genes were upregulated (54.53%). Under combined stress, 1976 DEGs were identified (Y79_96 vs. Y79_96HT), with 1072 genes downregulated (54.25%) and 904 genes upregulated (45.75%) (Fig. 2d).

To confirm that the transcriptome data were accurate, 12 DEGs were analyzed via quantitative reverse transcription PCR (qRT-PCR). The expression patterns of all DEGs were largely consistent between the qRT-PCR results and the RNA-seq data (Figure S2), validating the RNA-seq dataset for further analysis.

### KEGG pathway and GO analyses

**Fig. 3:**
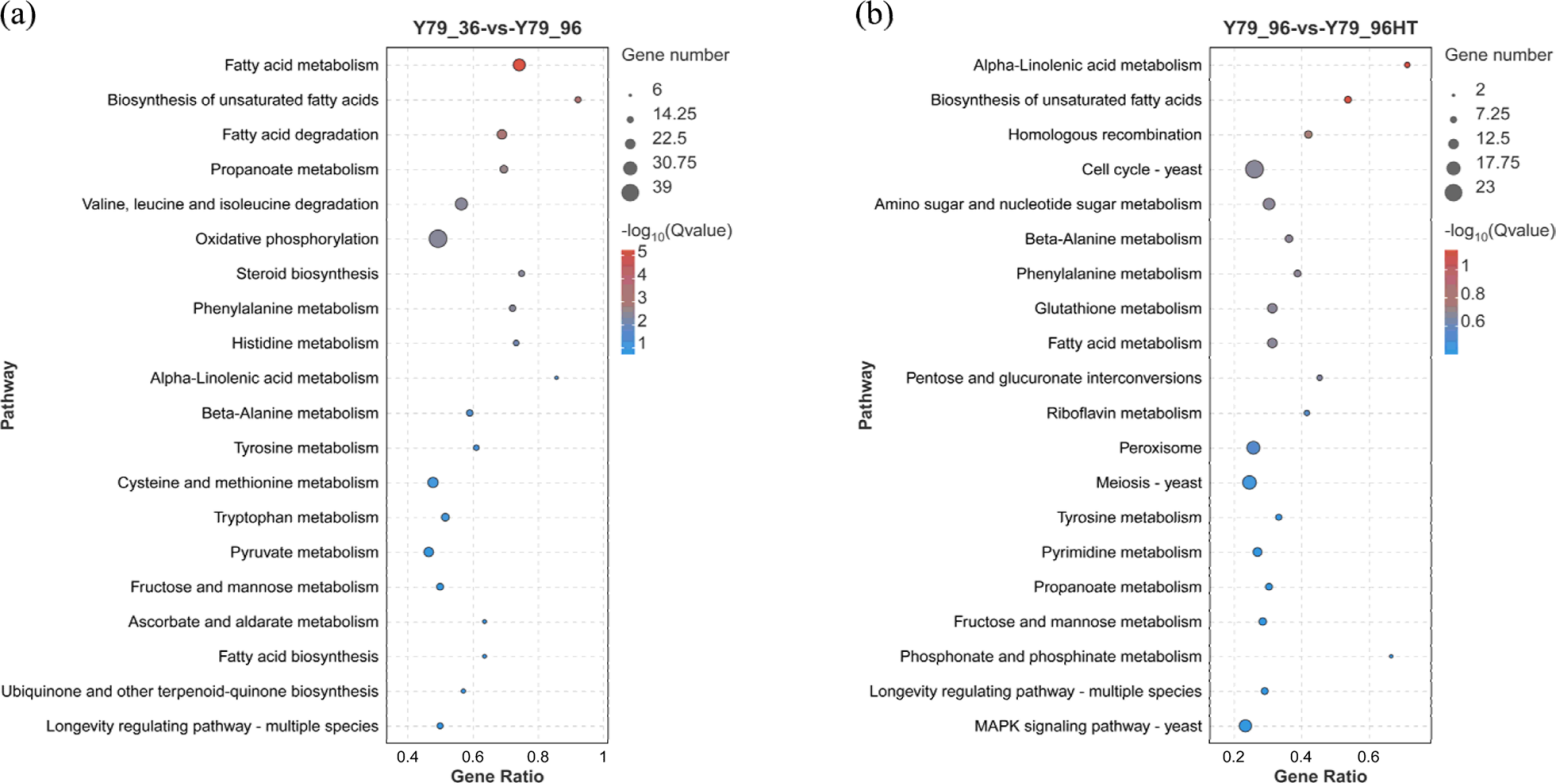
Enrichment analysis of the KEGG functional pathways. **a** Top 20 functional pathways associated with glucose starvation stress. **b** Top 20 functional pathways associated with combined stress. The color of the bubble indicates the enrichment degree of the pathway

To elucidate the response pathways of the YM25079 strain to both types of stress, functional enrichment and annotation analyses of DEGs were conducted using the Kyoto Encyclopedia of Genes and Genomes (KEGG) and Gene Ontology (GO) databases. In the KEGG analysis, the DEGs involved 115 and 110 pathways under glucose starvation and heat stress, respectively (Table S3). Regarding glucose starvation, the top 20 pathways included amino acid metabolism (ko00360, ko00340, ko00350, ko00270, ko00410, and ko00380), carbohydrate metabolism (ko00640, ko00620, ko00051, and ko00053), lipid metabolism (ko01040, ko00071, ko00100, ko00592, and ko00061), metabolism of cofactors and vitamins (ko00130), energy metabolism (ko00190), and aging (ko04213), among others (Fig. 3a). Similarly, regarding combined stress, the top 20 pathways included amino acid metabolism (ko00360 and ko00350), metabolism of other amino acids (ko00410, ko00480, and ko00440), carbohydrate metabolism (ko00520, ko00040, ko00640, and ko00051), cell growth and death (ko04111 and ko04113), lipid metabolism (ko00592 and ko01040), signal transduction (ko04011), and aging (ko04213), among others (Fig. 3b).

For the comprehensive functional characterization of the total DEGs, GO analysis was performed. The results analysis showed that the genes were primarily enriched in cellular process, metabolic process, binding, catalytic activity, cellular anatomical entity, protein-containing complex in biological process (BP), molecular function (MF), and cellular component (CC) (Figure S3). Under glucose starvation, in the BP category, the most significantly enriched pathways were transmembrane transport (GO:0055085), small-molecule metabolic process (GO:0044281), and organic acid metabolic process (GO:0006082). In the MF category, the most significantly enriched terms were related to oxidoreductase activity (GO:0016491), transmembrane transporter activity (GO:0022857), and xenobiotic transmembrane transporter activity (GO:0042910). In the CC category, the most enriched terms were intrinsic component of the plasma membrane (GO:0031226), integral component of the plasma membrane (GO:0005887), and inner mitochondrial membrane protein complex (GO:0098800) (Table S4). Under combined stress, the pathways most significantly enriched in BP, MF, and CC were associated with cellular amino acid catabolic process (GO:0009063), alpha-amino acid catabolic process (GO:1901606), organic acid catabolic process (GO:0016054), protochlorophyllide reductase activity (GO:0016630), oxidoreductase activity (GO:0016616, GO:0016614), glutathione transferase activity (GO:0004364), integral component of the plasma membrane (GO:0005887), intrinsic component of the plasma membrane (GO:0031226), and synaptonemal structure (GO:0099086)(Table S5).

These results indicated that glucose starvation and combined stress have distinct effects on the YM25079 strain. Under glucose starvation, membrane transport, oxidoreductase activity, and small-molecule metabolic processes are the most active, whereas under combined stress, organic acid catabolism, antioxidant capacity, and plasma membrane composition are the most affected.

### Nontargeted metabolomic analysis of the YM25079 strain in response to heat stress and glucose starvation stress

**Fig. 4:**
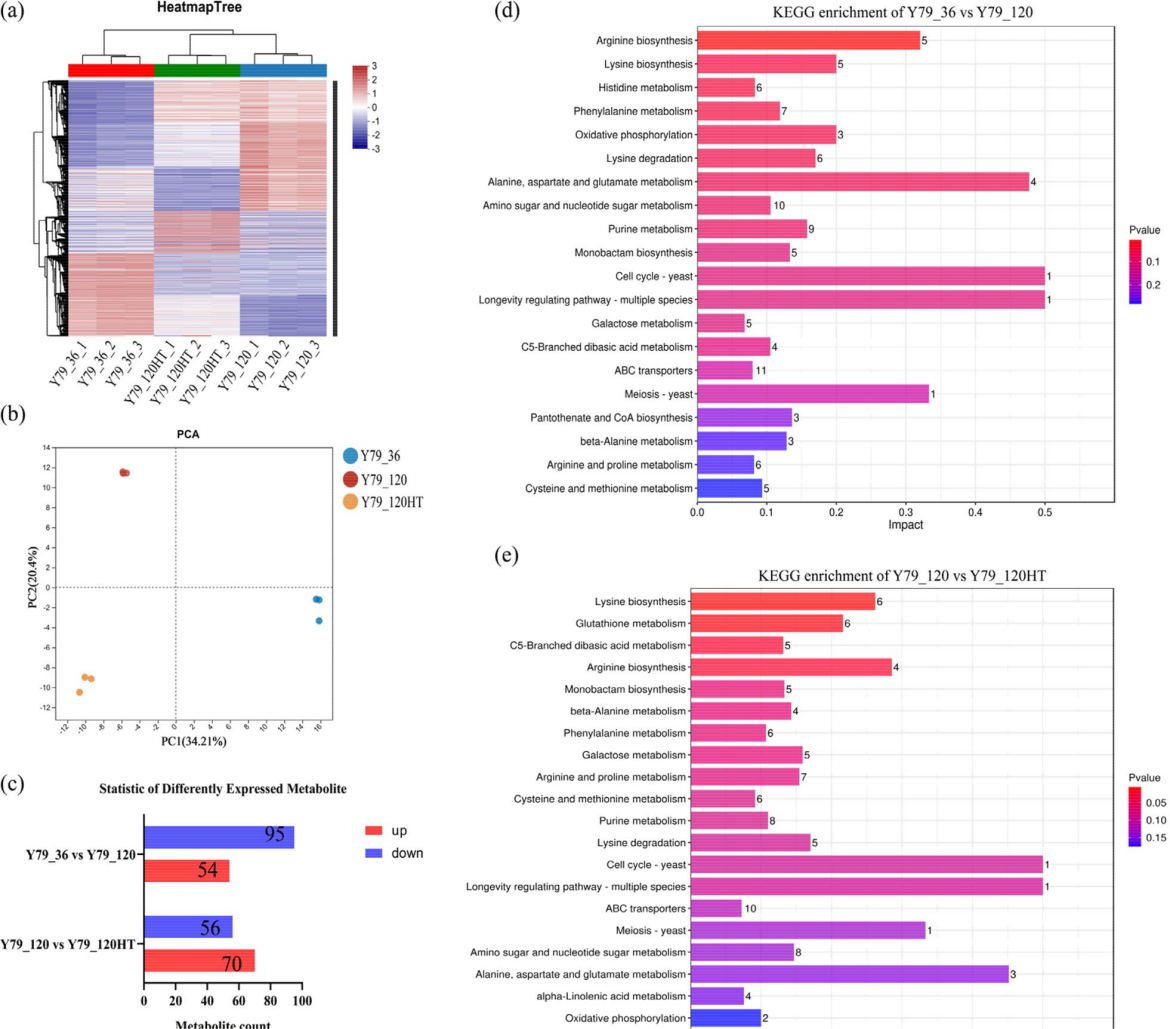
General overview of DAMs in YM25079 after heat stress and glucose starvation stress. **a** Correlation heatmap analysis. **b** PCA score scatter plots. **c** Bar chart of the DAMs. **d** The top 20 enriched KEGG terms related to glucose starvation stress. **e** The top 20 KEGG enrichment terms for combined stress. Note: Y79 represents YM25079, 36 and 120 represent the culture time, and HT represents heat stress

To elucidate the metabolic changes in YM25079 induced by glucose starvation and combined stress, nontargeted metabolomics analysis was conducted via liquid chromatography-tandem mass spectrometry (LC–MS/MS) on samples from two groups cultured for 120 h (Y79_120 and Y79_120HT) and a control group cultured for 36 h (Y79_36). Correlation heatmap analysis revealed the effect of different treatments on metabolite accumulation in YM25079, with good reproducibility recorded among samples within each group (Fig. 4a). PCA revealed significant differences between Y79_36, Y79_120 and Y79_120HT, enabling further analysis of differential metabolites (Fig. 4b). A total of 418 metabolites were detected in the total ion chromatogram (TIC), with the most abundant categories being carboxylic acids and their derivatives (19.38%), fatty acyls (9.09%), and organooxygen compounds (8.85%) (Table S6).

Additionally, 149 and 126 differential metabolites were identified under glucose starvation (Y79_36 vs. Y79_120) and combined stress (Y79_120 vs. Y79_120HT), respectively (Fig. 4c), with 60 and 58 metabolic pathways enriched through KEGG pathway analysis (Table S7). Pathways such as amino acid metabolism (sce00220, sce00300, sce00360, sce00310, sce00250, sce00410, sce00330, and sce00270), carbohydrate metabolism (sce00520 and sce00052), the longevity regulating pathway (sce04213), and ABC transporters (sce02010) were significantly enriched under both conditions. In contrast, glutathione metabolism and alpha-linolenic acid metabolism were significantly enriched only under combined stress (Figs. 4d and e). Among these differentially accumulated metabolites (DAMs), upregulated metabolites and their corresponding genes probably play key roles in the response of YM25079 to combined stress. Overall, these results showed that glucose starvation and heat stress have various effects on *Rhodotorula* cells, including but not limited to amino acid metabolism, carbohydrate metabolism, energy metabolism, and lipid metabolism.

### Integrative analysis of the transcriptome and metabolome

**Fig. 5:**
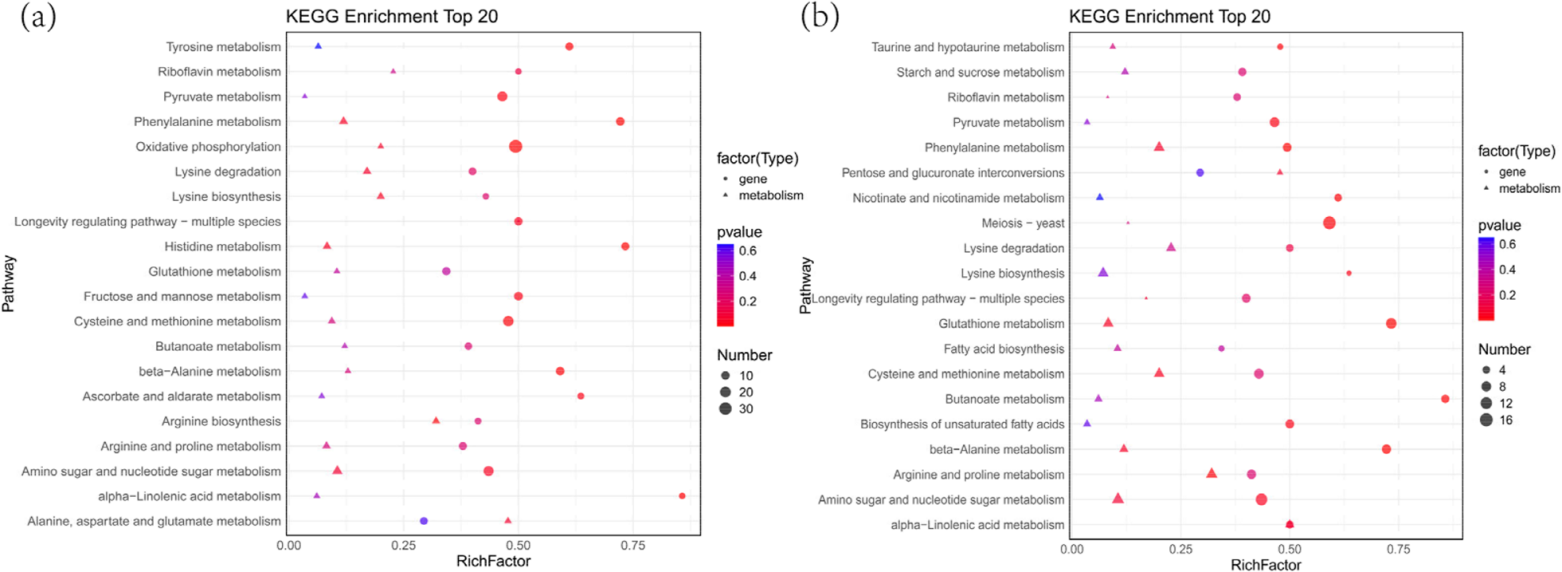
**a** The top 20 KEGG pathways enriched in DEGs and DAMs associated with glucose starvation stress. **b** The top 20 KEGG pathways enriched in DEGs and DAMs associated with combined stress. The X-axis indicates the enrichment score of the DEGs and DAMs. The p-value is indicated via a color scale; the size of the dots and triangles indicates the number of DEGs and DAMs mapped in each pathway, respectively

To elucidate the regulatory mechanisms by which the effects of heat stress and glucose starvation promote carotenoid biosynthesis, a comprehensive transcriptomic and metabolomic analysis was conducted. The KEGG analysis revealed that under glucose starvation, DEGs and DAMs were jointly enriched in 58 pathways, whereas under combined stress, 52 pathways were jointly enriched (Table S8). These metabolites and genes were involved mainly in amino acid metabolism, lipid metabolism, carbohydrate metabolism, energy metabolism, and terpenoid biosynthesis pathways. The most enriched pathways included phenylalanine metabolism, amino sugar and nucleotide sugar metabolism, the longevity regulating pathway, alpha-linolenic acid metabolism, pyruvate metabolism, glutathione metabolism, and cysteine and methionine metabolism (Fig. 5). These findings suggested that these metabolic pathways could associated with heat stress and glucose starvation stress.

**Fig. 6:**
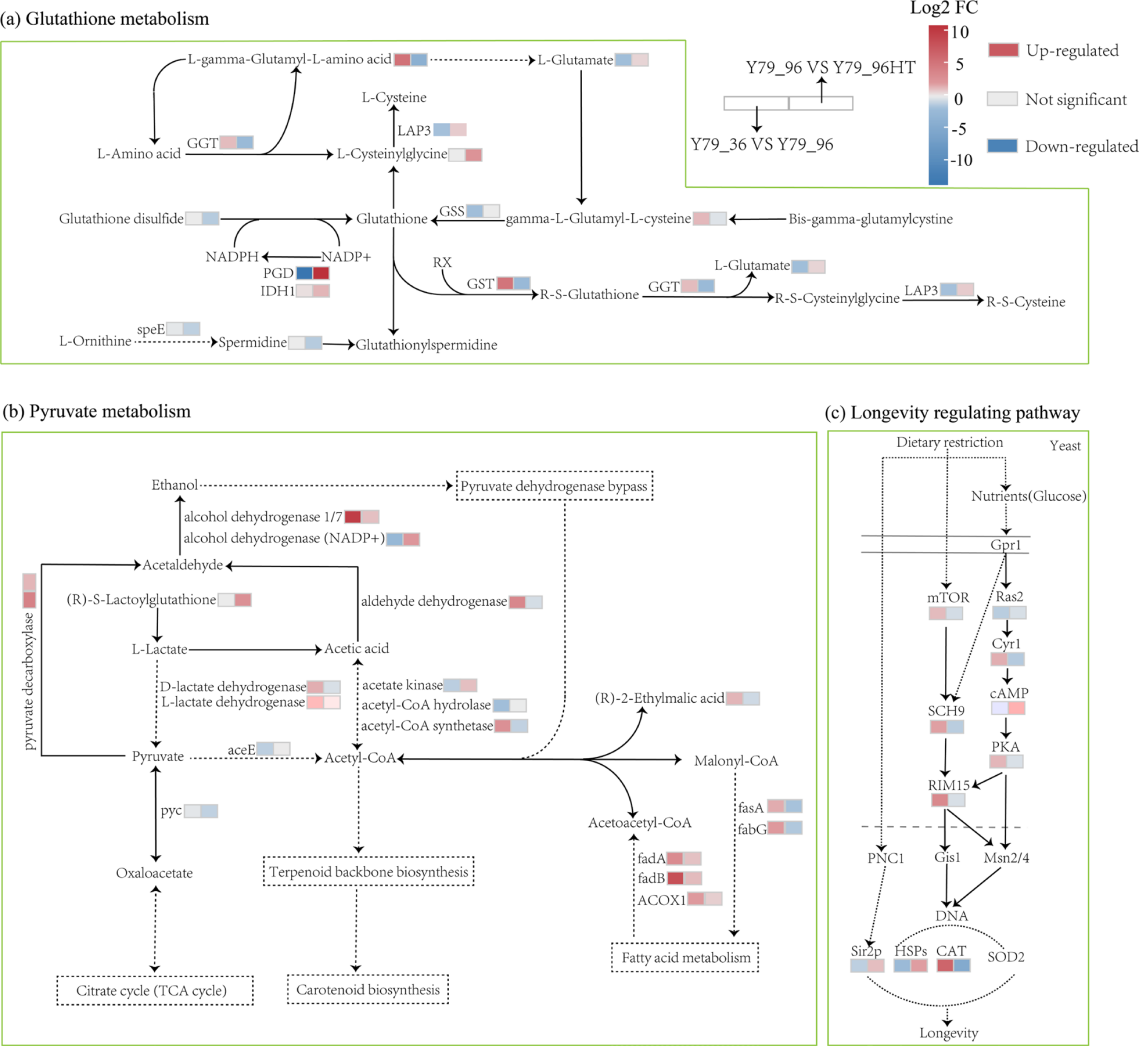
Changes in the main metabolic pathways in YM25079 after heat stress and glucose starvation stress. Based on the data generated by the KEGG database and with some modifications, a path was established. Dashed arrows indicate the simplified pathways. The colors of rectangles indicate significances, which are presented as a color scale

Further analysis revealed that the longevity regulatory pathway, glutathione metabolism, and pyruvate metabolism may play key roles under combined stress, with nine DEGs and one DAM, nine DEGs and six DAMs, and 12 DEGs and two DAMs associated with these pathways, respectively (Fig. 6). These findings indicated that YM25079 primarily regulates its response to the combined effects of glucose starvation and heat stress through the longevity-regulating pathway, followed by the non-enzymatic antioxidant mechanism (glutathione metabolism) and the consumption of acetyl-CoA (pyruvate metabolism). These pathways could help improve the tolerance of YM25079 to the effects of heat stress and glucose starvation stress.

### Analysis of the weighted gene and metabolite coexpression network

**Fig. 7:**
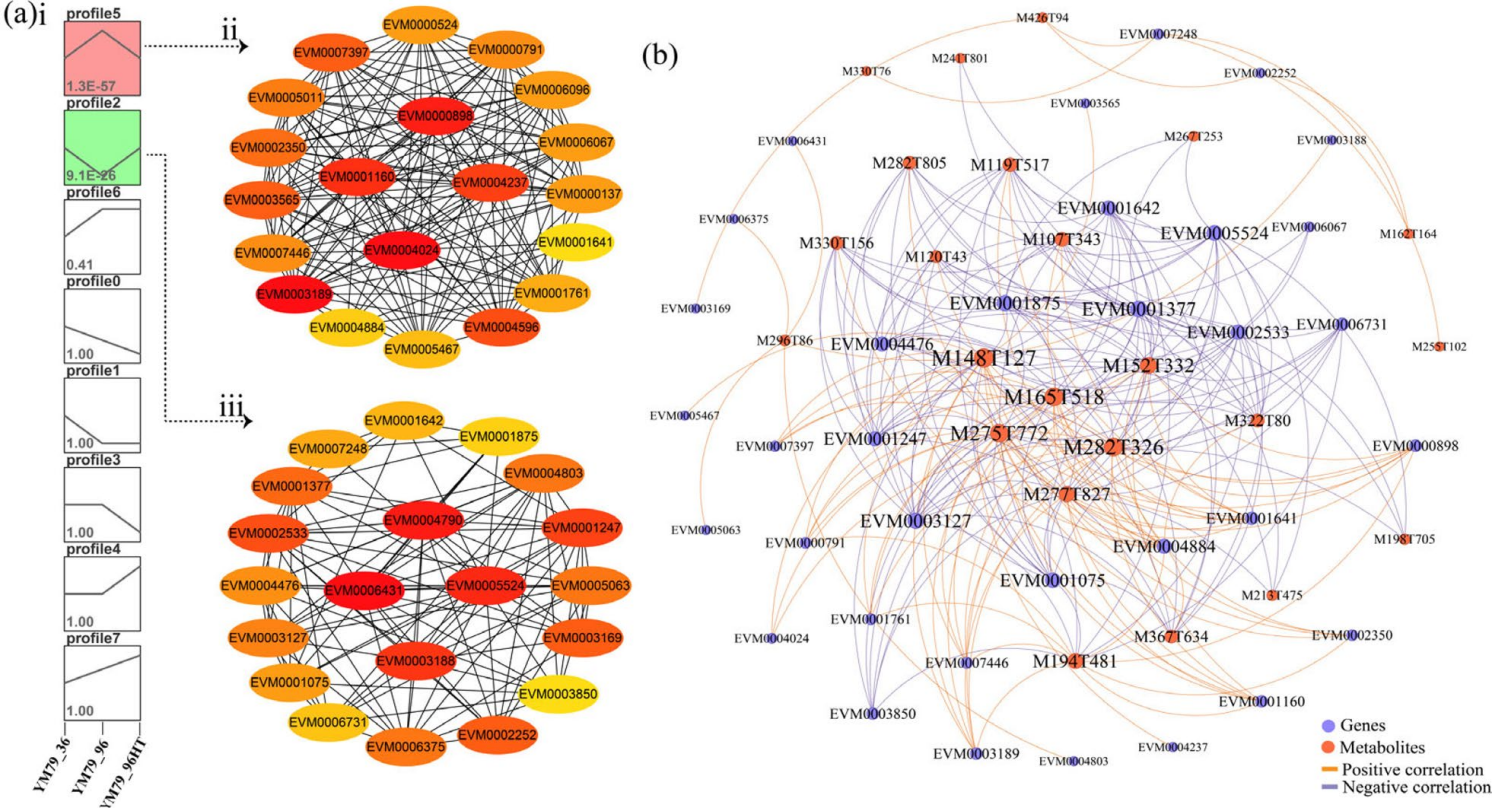
Analysis of the weighted gene and metabolite coexpression network. **a** Analysis of weighted genes. (i) Trend analysis of DEGs under heat stress and starvation stress. (ii) Top 20 hub genes for Trend 5. (iii) The top 20 hub genes for Trend 2. **b** Coexpression network of genes and metabolites. Genes and metabolites are represented by dots of different colors

To identify the key genes and metabolites in YM25079 under the effects of glucose starvation stress and heat stress, trend analysis was performed on the DEGs and DAMs selected from the stress conditions to determine the number of important candidate genes (Y79_36 vs. Y79_96 vs. Y79_96HT) and metabolites (Y79_36 vs. Y79_120 vs. Y79_120HT). The trend analysis grouped the DEGs into eight clusters, with Clusters 5 and 2 being significantly enriched, containing 360 and 284 genes, respectively (Fig. 7ai). Using the STRING database, a PPI network was constructed to illustrate the interactions between the genes within the enriched clusters. PPI network analysis revealed 91 nodes and 542 edges for Cluster 5 and 92 nodes and 635 edges for Cluster 2 (Table S9). The top 500 PPI networks, which were based on the combined score, were imported into Cytoscape. Using the CytoHubba plugin and degree centrality algorithms, the top 20 hub genes in each cluster were identified. The genes labeled red showed higher interaction scores (Figs. 7ii and 7iii).

Similarly, the DAMs were grouped into eight clusters, with Clusters 1 and 2 being significantly enriched, containing 20 and 14 metabolites, respectively (Figure S4). To determine the correlations between the 40 genes and 34 metabolites involved in the response of YM25079 to the effects of heat stress and glucose starvation stress, an interaction network was constructed using the Gephi v3.8.2 software based on the criteria of a PCC ≥|0.9| and p < 0.01 (Figs. 7b). The results revealed that 34 genes were correlated with 23 metabolites, with six metabolites, including L-methionine (M148T127), guanine (M152T332), m-coumaric acid (M165T518), Shogaol (M275T772), 2’-O-methyladenosine (M282T326), and straridonic acid (M277T827), identified as hub metabolites. These genes and metabolites probably play key roles in the response of YM25079 to the combined effects of glucose starvation and heat stress.

## Discussion

In general, the production of carotenoids as secondary metabolites is not considered essential for the growth of most microorganisms. Its biosynthesis primarily serves as a protective mechanism in response to environmental stress [28]. Therefore, understanding how to economically increase carotenoid production under environmental stress conditions is considered to be a future development trend. Identifying the key factors that influence cell growth and the production of valuable products is equally important. In this study, high temperature promoted the early growth of *R. glutinis* YM25079 (cultured at 30 °C for 24 h), possibly by stimulating mitotic recombination and accelerating the cell cycle [29]. However, prolonged exposure to high temperatures adversely affects the reproductive capacity of the cells, leading to excessive ROS production and cell apoptosis [30], which is consistent with our experimental results (Figs. 1a and S1b). In response to oxidative stress, microorganisms activate ROS-scavenging systems to enhance antioxidant capacity, which mainly consist of enzymes antioxidants (superoxide dismutase (SOD), catalase (CAT), etc.) and non-enzymatic antioxidants(glutathione, trehalose and carotenoids, etc.) [31]. Some studies have reported a correlation between carotenoid biosynthesis and fluctuations in intracellular oxidative stress levels [32–34]. Therefore, the ROS generated under heat stress may have contributed to the accumulation of carotenoids in YM25079 during the initial stage (cultured at 30 °C for 24–72 h). However, this behavior was enhanced when the strain entered a state of glucose starvation. The relationships between the combined effects of heat stress and glucose starvation stress on carotenoid biosynthesis and the underlying mechanisms need to be determined.

Prolonged exposure to high temperatures disrupts mitochondrial function, induces protein folding stress, and promotes lipid peroxidation of the cell membrane, leading to a significant increase in intracellular ROS levels [35, 36]. Therefore, maintaining membrane lipids and removing excess ROS are considered crucial for cell survival. However, under heat stress, enzymes involved in the synthesis, degradation, and conversion of glutathione, such as γ-glutamyltransferase/glutathione hydrolase (EVM0006641, log2 FC =  − 2.19) and glutathione S-transferase (EVM0000788, log2 FC =  − 2.36), reduce its activity. Additionally, the synthesis precursors of glutathione, such as glutathione disulfide (M613T149_1, log2 FC =  − 1.33) and gamma-L-glutamyl-L-cysteine (M233T131, log2 FC =  − 0.32), were downregulated. Prolonged heat stress can impair CAT activity [37], hindering its normal function, whereas SOD activity can increase antioxidant capacity [38, 39]. In this study, the expression of the CAT gene (EVM0001641, log2 FC =  − 4.91) was downregulated without significant changes in SOD activity. SOD and CAT serve as the primary defense mechanisms for microorganisms against oxidative stress. When antioxidant enzyme activities are suppressed by heat stress, the non-enzymatic antioxidant system is activated, leading to the overproduction of certain antioxidants, such as carotenoids and polysaccharides [40]. The transcript levels of key genes responsible for carotenoid biosynthesis were not upregulated during carotenoid overproduction, which was consistent with the findings of several previous studies [41]. Both low-temperature and high-temperature stresses affect the activity of carotenoid synthesis genes (CrtYB, and CrtI) in microorganisms [42, 43]. Although the expression of carotenoid biosynthesis genes did not significantly change under high-temperature treatment in YM25079, their enzyme activity may have been appropriately stimulated to ensure that carotenoids were produced in large amounts to counteract oxidative stress. Moreover, certain amino acids with antioxidant properties, such as methionine (M148T127, log2 FC = 5.26) and histidine (M156T83, log2 FC = 1.55), accumulated. Reducing the ratio of unsaturated fatty acids (UFAs) to saturated fatty acids (SFAs) in the cell membrane enhances heat tolerance but reduces membrane fluidity [44], as UFAs are more prone to peroxidation under heat stress [45]. Therefore, significant enrichment of the UFA biosynthesis pathway (ko00592 and ko01040) was observed, while stearoyl-CoA desaturase (EVM0007632, log2FC =  − 1.35), a key gene involved in UFA biosynthesis, was downregulated. The cell membrane maintains a certain degree of fluidity to improve cell survival and carotenoid accumulation not only enhances membrane fluidity but is also strongly correlated with UFA production [46]. Exogenous supplementation with UFAs can increase the carotenoid content, which is consistent with the hypothesis that more carotenoids are required to maintain the unsaturated state of UFAs [47]. Additionally, the downregulation of lipid biosynthesis-related genes such as fatty acid synthase subunit α (FAS2, EVM0004884, log2 FC =  − 1.72), 3-oxoacyl- [acyl-carrier protein] reductase (fabG, EVM0004680, log2 FC =  − 1.47), glycerol-3-phosphate O-acyltransferase 1/2 (EVM0002078, log2 FC =  − 1.05), glycerol 2-dehydrogenase (EVM0000565, log2 FC =  − 1.17) was consistent with the results of reduced lipid content observed in this study (Figure S1c). Overall, YM25079 generated high levels of ROS under heat stress, and its enzymatic antioxidant capacity was compromised. Heat tolerance was improved by increasing carotenoid accumulation and regulating energy balance and lipid membrane repair.

The effect of combined or sequential stressors ranges from neutral and additive effects to synergistic effects, sometimes resulting in novel and unpredictable responses [48, 49]. In this study, the YM25079 strain was found to adapt primarily to glucose starvation by coordinating multiple biological processes, particularly lipid, amino acid, carbohydrate, and energy metabolism (Figs. 3a and 4d). However, the regulatory mechanisms that enable adaptation to glucose starvation also had new effects on the heat tolerance of the strain. Under starvation conditions, phosphoenolpyruvate carboxykinase (PCK, EVM0003641, log2 FC = 1.80) is activated, allowing glucose synthesis from substrates such as succinate, pyruvate, or glycine [50]. Additionally, regulatory genes such as glutamate dehydrogenase (GDH2, EVM0005815, log2 FC = 3.91) and aspartate aminotransferase (AST, EVM0003168, log2 FC = 1.12) are upregulated, promoting the entry of amino acids into energy metabolism pathways to maintain cell survival and growth [51]. This also reduces the antioxidant capacity provided by amino acids and the precursors available for glutathione synthesis [52]. Moreover, the decrease in NADPH levels caused by glucose deficiency further weakens the ability of glutathione reductase (GSR) to convert oxidized glutathione (GSSG) back to its reduced form GSH [53, 54]. Fatty acid β-oxidation was found to increase carotenoid production by providing acetyl-CoA and energy to the mevalonate (MVA) pathway [41], regulated genes such as acyl-CoA oxidase (ACOX, EVM0002438, log2 FC = 2.00), acetyl-CoA acyltransferase (fadA, EVM0007108, log2 FC = 3.18), and 3-hydroxyacyl-CoA dehydrogenase (fadB, EVM0002939, log2 FC = 8.35), were upregulated, improving the ability of the cell to metabolize lipids [55]. As acetyl-CoA is a common precursor for the carotenoid and lipid biosynthesis pathways, the allocation of acetyl-CoA is considered critical for promoting downstream carotenoid production. The upregulation of genes such as pyruvate decarboxylase (EVM0004263, log2 FC = 4.02), aldehyde dehydrogenase (EVM0001820, log2 FC = 3.53), and alcohol dehydrogenase (EVM0007554, log2 FC = 9.89) enhances pyruvate dehydrogenase bypass (PDH bypass), which directly generates acetyl-CoA for lipid or terpene biosynthesis [56]. Moreover, carotenoid biosynthesis in *R. glutinis* is influenced by the carbon-to-nitrogen (C/N) ratio of the medium [21, 57], and acetyl-CoA is more likely to be diverted to carotenoid production rather than lipid synthesis under carbon starvation. These results suggest that the metabolic flux of acetyl-CoA may have changed, with more flux directed toward terpenoid biosynthesis or energy metabolism pathways rather than lipid biosynthesis, this may also be one of the reasons why the carotenoid content increased but its biosynthesis genes (CrtYB, and CrtI) did not change significantly. Moreover, autophagy-related genes, such as ATG8 (EVM0001887, log2 FC = 1.32), ATG9 (EVM0001982, log2 FC = 1.20), and ATG11 (EVM0004355, log2 FC = 1.28), are upregulated to help cells adapt to nutrient-limited environments and provide necessary nutrients [58, 59]. Signal transduction pathways such as the mTOR, PKA, and Sir2P pathways regulate cellular metabolism and physiological states, enabling the cell to enter energy-saving and self-protection modes, thus reducing energy consumption [60–62] (Fig. 6c). Overall, under glucose starvation, YM25079 enhanced nutrient acquisition, directing resources to energy metabolism pathways or precursors for synthesizing secondary metabolites, further enhancing stress resistance. Similar metabolic changes occur in other microorganisms [14, 55, 63]. Additionally, glucose starvation affects the antioxidant capacity of a strain. As compensation, YM25079 may increase the utilization of acetyl-CoA as a substrate for terpenoid biosynthesis or energy metabolism while reducing its consumption in lipid biosynthesis.

Along with carotenoids, several other metabolites may play important roles in the response of YM25079 to stress. Among the key metabolites identified (Fig. 7b), secondary metabolites such as Sequoyitol (M194T481, log2 FC = 1.48), m-coumaric acid (M165T518, log2 FC = 0.56), and Shogaol (M275T772, log2 FC = 2.97) were shown to help counteract oxidative stress by scavenging excess ROS [64–66]. Lipid products, such as SFAs, including stearidonic acid (M277T827, log2 FC = 1.86) and pentadecanoic acid (M241T801, log2 FC = 1.36), can increase the heat resistance of the cell membrane [67]. The integrity and fluidity of the membrane are maintained by sphingosine (M282T805, log2 FC = 1.38) and dodecanedioic acid (M213T475, log2 FC = 2.69) [68, 69], further assisting cells in adapting to adverse environmental conditions by regulating energy balance. L-methionine (M148T127, log2 FC = 5.62), selenomethionine (M198T705, log2 FC =  − 0.47), and nicotinamide riboside (M255T102, log2 FC = 1.77) contribute to the synthesis of glutathione peroxidase, which protects cells from oxidative damage [70–72]. Additionally, several genes closely related to these metabolites, including glyceraldehyde-3-phosphate dehydrogenase (EVM0001642, log2 FC = 4.32), are upregulated under combined stress, which not only accelerates glycolysis to provide energy but also promotes the expression of heat-induced genes, thus increasing heat resistance [73]. Catalase (EVM0001641, log2 FC =  − 4.91), an important antioxidant enzyme reported to inhibit its activity, promotes carotenoid biosynthesis in *Dactylococcus dissociates* [74]. Genes involved in the regulation of the cell cycle, such as DNA repair and recombination proteins (EVM0006731, log2 FC = 2.39) and the DNA repair protein RAD51 (EVM0001377, log2 FC = 3.99), respond quickly to DNA damage induced by heat stress; these proteins collectively maintain cell survival and genome stability [75]. These core metabolites and genes may play a leading role in the response of YM25079 to the effects of heat stress and glucose starvation.

## Conclusions

In this study, the effects of combined stress from heat and glucose starvation on carotenoid biosynthesis in YM25079 were investigated. The results of transcriptomic and metabolomic analyses revealed that the effects of glucose starvation and heat stress on promoting carotenoid biosynthesis appeared to be additive, as the combined stress further increased the ROS levels, reduced the enzymatic antioxidant capacity and promoted carotenoid biosynthesis relative to single stress. The key responses of YM25079 to multiple stresses include regulation of the cell cycle, regulation of energy metabolism, maintenance of membrane integrity, and promotion of non-enzymatic antioxidant activity. Carotenoids play crucial roles in eliminating ROS, increasing tolerance to oxidative stress, and maintaining the structure and function of the cell membrane. In this study, we elucidated the regulatory mechanisms underlying increased carotenoid biosynthesis under the combined stress of heat and glucose starvation and also identified key candidate genes and metabolites, providing deeper insights into the molecular and metabolic basis of YM25079. In addition, improving the heat resistance of YM25079—for example, through the screening of heat-adapted strains—may further enhance carotenoid production and mitigate the trade-off between biomass and carotenoid yield. This will be a key objective of our future research.

## Supplementary Information

Supplementary file 1.

## Data Availability

The RNA-seq data generated in this study have been deposited on the National Center for Biotechnology Information platform (NCBI) under the BioProject ID: PRJNA1091310.

## Abbreviations

BP: Biological process
CAT: Catalase
CC: Cellular component
DAMs: Differentially accumulated metabolites
DEGs: Differentially expressed genes
GO: Gene Ontology
KEGG: Kyoto Encyclopedia of Genes and Genomes
MF: Molecular function
PCA: Principal component analysis
qRT-PCR: Quantitative reverse transcription PCR
ROS: Reactive oxygen species
SOD: Superoxide dismutase
PCC: The Pearson correlation coefficient
