# Supplementary material for: Integrated transcriptomics and metabolomics analysis reveal the regulatory mechanisms underlying the combined effects of heat and glucose starvation on carotenoid biosynthesis in Rhodotorula glutinis YM25079
Source: Biotechnol Biofuels Bioprod. 2025 Jul 10;18:71. doi: 10.1186/s13068-025-02678-7 (PMC12243306; doi:10.1186/s13068-025-02678-7)
Supplement: Supplementary file 1 [file 13068_2025_2678_MOESM1_ESM.zip › Supplementary figures.docx]

**Supporting Information**

**Integrated transcriptomics and metabolomics analysis reveal the regulatory mechanisms underlying the combined effects of heat and glucose starvation on carotenoid biosynthesis in *Rhodotorula glutinis* YM25079**

Xingyu Huang, Caina Guo, Xiaolan Huang, Meixia He, Jingdie Fan, Yuan Chen, Jingwen Qiu* and Qi Zhang*

**Affiliation:** Faculty of Life Science and Technology, Kunming University of Science and Technology, Kunming, 650500, Yunnan, P.R. China

***Correspondence:** qiujingwenrr@outlook.com (J.Q.); qzhang37@kust.edu.cn (Q.Z.)


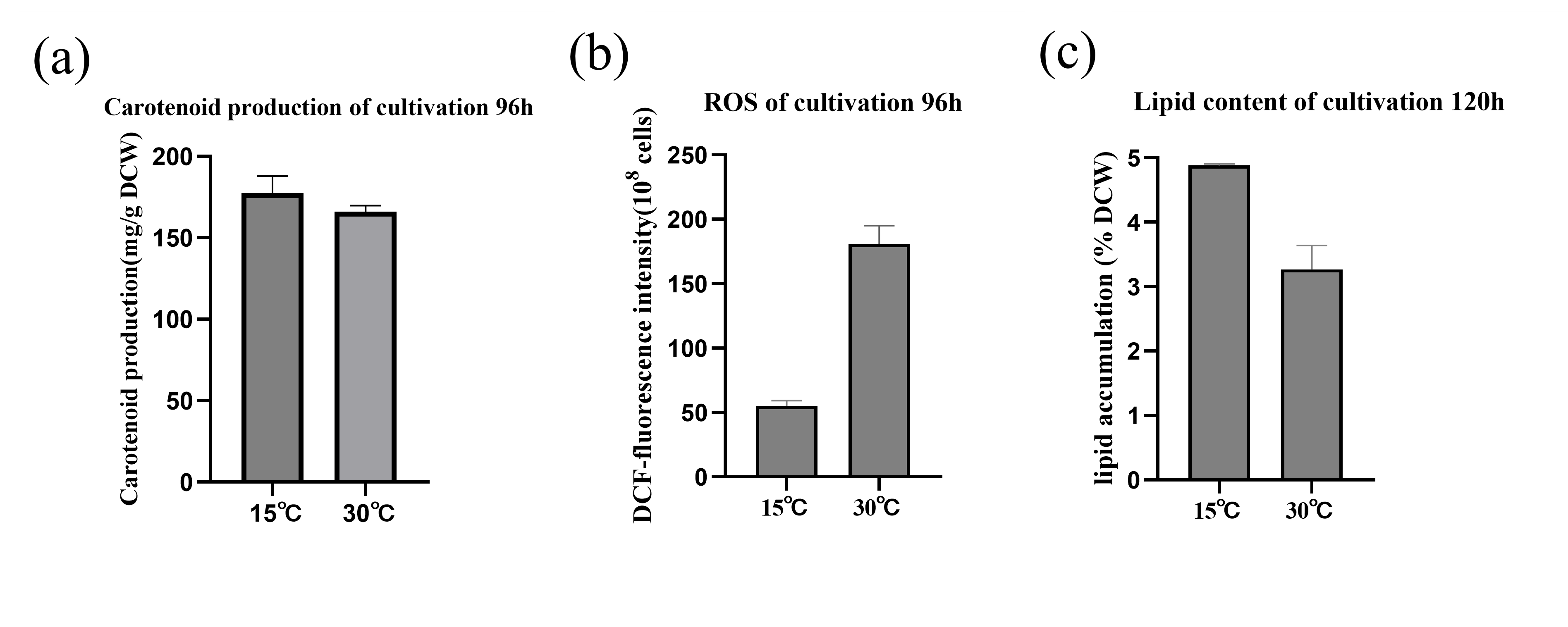


Figure S1. (a)Carotenoid production after heat stress at 96 h. (b) Effects of heat stress on ROS at 96 h. (c) Lipid content after heat stress at 120 h. The data are presented as the mean ±standard deviation of triplicate samples.

Figure S2. Quantitative RT-qPCR validations for RNA-seq data. The figure displays the average values ± standard deviation (SEM) of three independent experiments, with statistical differences denoted as *: 0.01 < p < 0.05, **: 0.001 < p < 0.01.


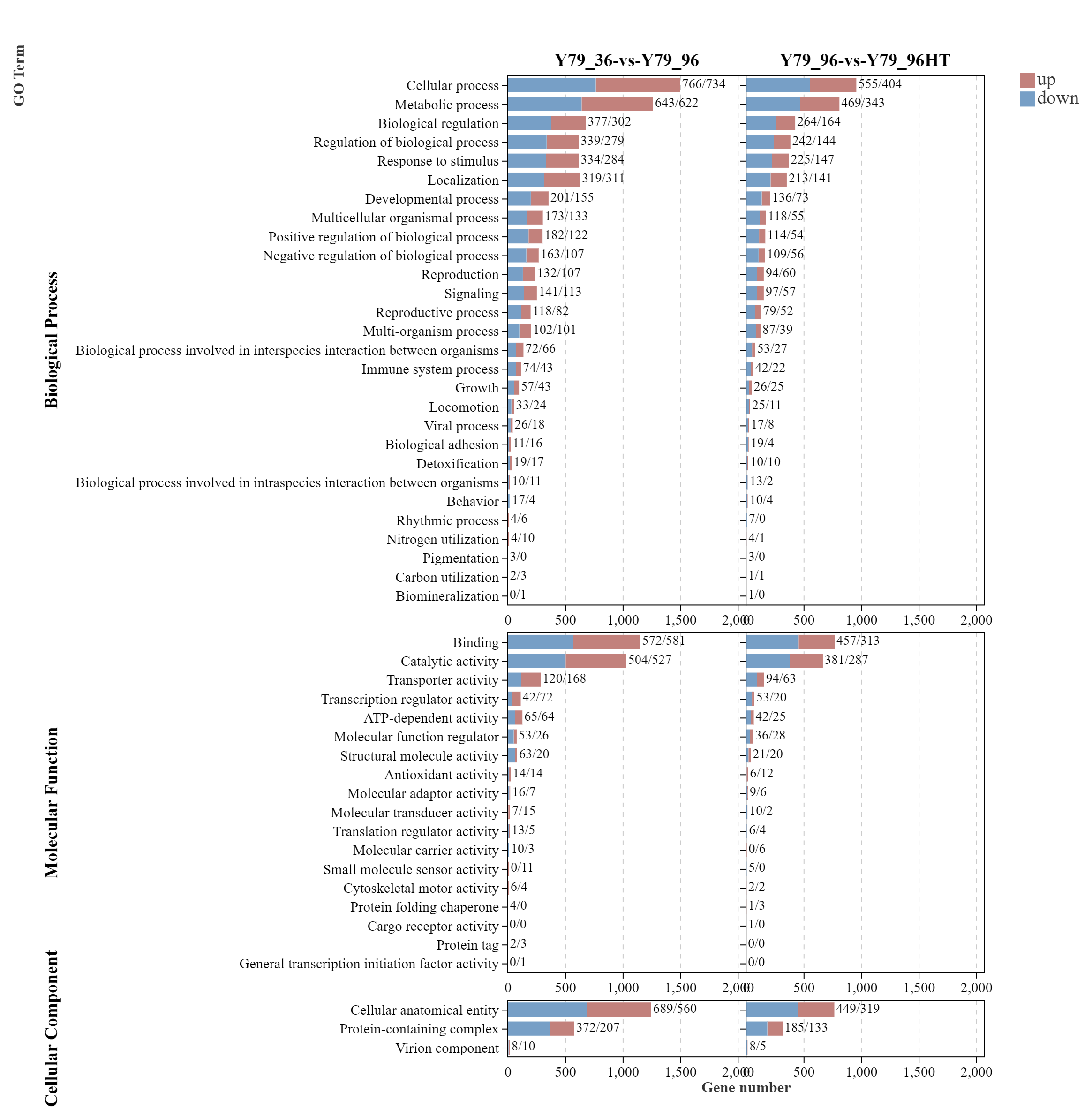


Figure S3. Functional annotation of DEGs based on gene ontology categorization.


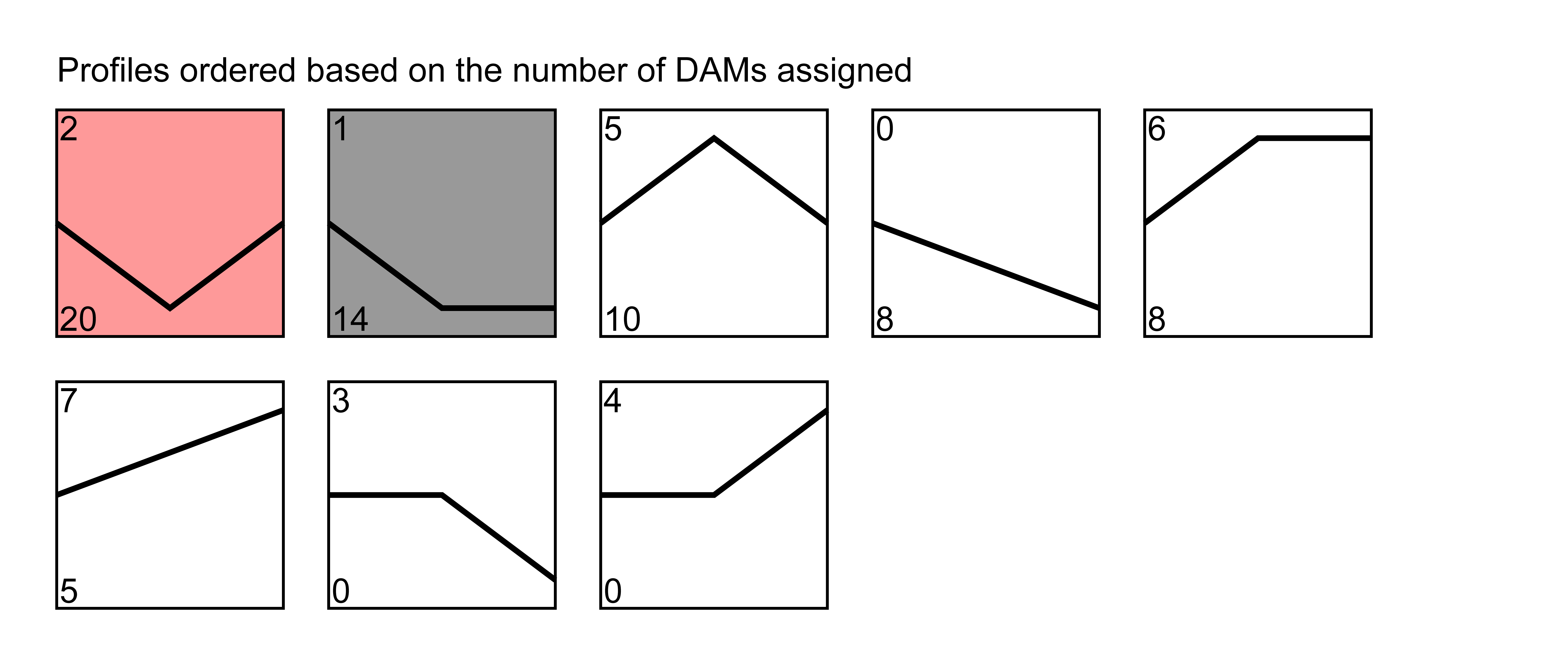


Figure S4. The trend analysis of DAMs
